# Clinical and psychometric correlates of binge eating behaviors during the COVID-19 pandemic

**DOI:** 10.1192/j.eurpsy.2023.1108

**Published:** 2023-07-19

**Authors:** V. Efstathiou, E. Samara, E. Efstathiou, K. Gkikas, K. Papazachos, P. Bali, E. Kaloudi, I. Giannopoulou, I. Michopoulos, A. Papadopoulou

**Affiliations:** 1 Psychology Department, National and Kapodistrian University of Athens; 2 Marketing and Communication Department, Athens University of Economics and Business; 3 Second Department of Psychiatry, National and Kapodistrian University of Athens, “Attikon” University General Hospital, Athens, Greece

## Abstract

**Introduction:**

Binge eating behaviors are associated with psychological, social, and biological factors, while it is suggested that they may be triggered by negative emotions, including depression and anxiety, and provide relief from them, which in turn may lead to reinforcement of such behaviors.

**Objectives:**

This study aimed to examine the eating habits and in particular the binge eating behaviors of a sample of adults during the COVID-19 pandemic, an unprecedented challenge for public health and communities worldwide with multi-level consequences on people’s lives.

**Methods:**

The sample consisted of 196 individuals residing in Greece aged 18 to 64 years (76.5% women), who completed an anonymous questionnaire from June to July 2021. This included the following psychometric instruments: Fear of COVID-19 Scale to assess the fear related to COVID-19, Rosenberg Self-esteem Scale to assess self-esteem, Depression Anxiety Stress Scale-21 to assess anxiety, depression and stress, Binge Eating Scale to assess binge eating behaviors, UCLA Loneliness Scale for the evaluation of the perceived feeling of loneliness and Reflective Functioning Questionnaire for the assessment of reflective functioning (i.e., the ability to understand human behavior in terms of underlying mental states).

**Results:**

The majority of participants (86.7%) reported that during the pandemic their diet was less healthy than before the pandemic onset, while almost half (46.4%) of the participants stated that they had experiences an episode of binge eating during the past 6 months, and 36.2% that they had used self-induced vomiting in order to control their weight. Of note, the results of a multiple regression analysis revealed that higher levels of fear of the pandemic as well as of depression were independently associated with higher binge eating, with women presenting higher mean scores in the Binge Eating Scale than men. Conversely, higher self-esteem appeared to be independently associated with lower binge eating levels, thus acting as a protective factor, whereas the remaining psychometric factors were not found statistically significantly related.

**Conclusions:**

In conclusion, the findings of the present study highlight the importance of identifying dysfunctional eating behaviors and related psychological factors that may potentially act as risk or protective factors, especially during the pandemic.

**Disclosure of Interest:**

None Declared

